# Efficacy and safety of single-dose artesunate plus sulfalene/pyrimethamine combined with praziquantel for the treatment of children with *Schistosoma mansoni* or *Schistosoma haematobium* in western Kenya: a randomised, open-label controlled trial

**DOI:** 10.1186/s13071-024-06359-6

**Published:** 2024-06-28

**Authors:** Charles O. Obonyo, Fredrick O. Rawago, Nicholas K. Makworo, Erick M. O. Muok

**Affiliations:** 1 Centre for Global Health Research, Kenya Medical Research Institute, Kisumu, Kenya; 2 Division of Vector-Borne and Neglected Tropical Diseases, County Department of Health, Migori, Kenya

**Keywords:** Schistosomiasis, Praziquantel, Artesunate plus sulfalene-pyrimethamine, Artemisinin-based combinations, *Schistosoma mansoni*, *Schistosoma haematobium*

## Abstract

**Background:**

Reliance on praziquantel for the treatment and control of schistosomiasis is likely to facilitate the emergence of drug resistance. Combination therapy targeting adult and juvenile schistosome worms is urgently needed to improve praziquantel efficacy and delay the potential development of drug resistance. We assessed the efficacy and safety of single-dose praziquantel combined with single-dose artesunate plus sulfalene-pyrimethamine in the treatment of Kenyan children with schistosomiasis.

**Methods:**

This was an open-label, randomised clinical trial involving 426 school-aged children (7–15 years old) diagnosed with *Schistosoma mansoni* (by Kato-Katz) or *S. haematobium* (by urine filtration). They were randomly assigned (1:1:1) to receive a single dose of praziquantel (40 mg/kg), a single dose of artesunate plus sulfalene-pyrimethamine (12 mg/kg artesunate) or combination therapy using a single dose of praziquantel (40 mg/kg) combined with a single dose of artesunate plus sulfalene-pyrimethamine (12 mg/kg artesunate). The primary outcome was cure and egg reduction rates at 6 weeks post-treatment in the available case population. Adverse events were assessed within 3 h after treatment.

**Results:**

Of the 426 children enrolled, 135 received praziquantel, 150 received artesunate plus sulfalene-pyrimethamine, and 141 received combination therapy. Outcome data were available for 348 (81.7%) children. For *S. mansoni*-infected children (*n *= 335), the cure rates were 75.6%, 60.7%, and 77.8%, and the egg reduction rates were 80.1%, 85.0%, and 88.4% for praziquantel, artesunate plus sulfalene-pyrimethamine, and combination therapy, respectively. For *S. haematobium*-infected children (*n* = 145), the corresponding cure rates were 81.4%, 71.1%, and 82.2%, and the egg reduction rates were 95.6%, 97.1%, and 97.7%, respectively. Seventy-one (16.7%) children reported mild-intensity adverse events. The drugs were well tolerated and no serious adverse events were reported.

**Conclusions:**

A single oral dose of praziquantel combined with artesunate plus sulfalene-pyrimethamine cured a high proportion of children with *S. haematobium* but did not significantly improve the treatment efficacy for either urinary or intestinal schistosomiasis. Sequential administration of praziquantel and artesunate plus sulfalene-pyrimethamine may enhance the efficacy and safety outcomes.

**Graphical abstract:**

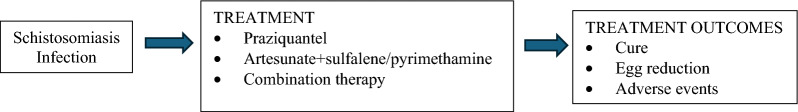

## Background

Human schistosomiasis is an infectious disease caused by trematode worms of the *Schistosoma* genus [1]. The three most common schistosome species infecting human populations include *Schistosoma mansoni, S. haematobium*, and *S. japonicum*. The global burden of schistosomiasis is estimated at 1.4–3.3 million disability-adjusted life years annually [2]. Over 90% of the 250 million people infected with schistosomiasis reside in sub-Saharan Africa, where *S. mansoni* and *S. haematobium* are the most prevalent species [3]. The high-risk groups for schistosomiasis include preschool- and school-aged children, with consequences that include anaemia, school absenteeism, impaired child growth, physical fitness, and impaired cognitive and intellectual development [4].

The main global schistosomiasis control strategy is preventive chemotherapy using praziquantel because it is effective against all the species of human schistosomiasis, it can be administered as a single oral dose, and it is affordable, safe, and partially effective [5, 6]. Praziquantel is effective against adult schistosome worms but less effective against the parasite’s juvenile stages (schistosomula) [7]. Reliance on praziquantel for wide-scale preventive treatment exerts high drug pressure and could favour the emergence of praziquantel-resistant parasites. Although clinical resistance to praziquantel has not yet been established, laboratory and community studies have shown reduced schistosome susceptibility [8–10]. New drugs are urgently needed to complement the use of praziquantel. Unfortunately, the schistosomiasis drug development pipeline is empty. A promising strategy to improve praziquantel efficacy and delay the potential development of drug resistance is combination therapy, in which two or more drugs with different mechanisms of action are administered together. Drug repurposing may be a low-risk, cost-effective, and time-saving approach for developing new treatments by identifying new indications for established drugs [11]. Possible candidates for anti-schistosomal combination therapy include praziquantel plus antimalarials such as mefloquine or artemisinin derivatives [12]. Artemisinin derivatives, such as artesunate, artemether, or dihydroartemisinin, are promising candidates for treating and preventing schistosomiasis [13, 14].

Artemisinin-based combination therapies (ACTs) are the most effective treatments for malaria [15]. Coincidentally, there is a large geographical overlap in sub-Saharan Africa's schistosomiasis and malaria co-endemic areas [16]. Artemisinin derivatives have been shown to have anti-schistosomal activity in vitro, in vivo, and in clinical trials [17, 18]. Artesunate or artemether alone is less effective than praziquantel, but significantly greater cure and egg reduction rates were observed when artesunate or artemether was combined with praziquantel compared to any of these medicines alone [19]. Unlike praziquantel, artemisinin derivatives are effective against juvenile schistosome worms [13]. Differences in the mechanism of drug action support a theoretical basis for combining praziquantel with an ACT for treating schistosomiasis. In high-transmission settings, combination therapy is synergistic, acting at two stages of the schistosome life cycle to cure the primary infection and block further transmission. In clinical trials, ACTs are less effective than praziquantel in treating human schistosomiasis [20–23]. Laboratory studies with animal models suggest that combined therapy using praziquantel and artemisinin derivatives may be an attractive novel treatment for human schistosomiasis [24, 25]. However, two studies that assessed the efficacy of praziquantel plus an ACT for treating schistosomiasis showed mixed results [26, 27].

It is unclear whether the dosing regimen influences the efficacy of ACTs against schistosomiasis. For instance, a significantly lower cure rate of 14% was observed with a 3-day regimen (once daily for 3 days) of artesunate plus sulfalene-pyrimethamine against *S. mansoni* compared to a cure rate of 44% with a 24-h treatment regimen against *S. haematobium* [21, 22]. Similarly, whether ACT doses used to treat malaria effectively cure schistosomiasis is unknown. If combination therapy using ACT plus praziquantel is found to be safe and effective, there could be concerns about the feasibility of a 3-day course of ACT for mass drug administration. This study aimed to assess the role of single-dose combination therapy (praziquantel combined with artesunate plus sulfalene-pyrimethamine) in the treatment of schistosomiasis in an area of *S. mansoni* and *S. haematobium* co-endemicity in western Kenya. For this study, we selected artesunate plus sulfalene-pyrimethamine because it is widely available as an ACT for malaria treatment, is co-formulated, is available for single daily dose therapy, has a well-established safety profile, and has been evaluated for the treatment of schistosomiasis with inconclusive results. The praziquantel-alone group was considered the control arm for all the treatment comparisons.

## Methods

### Study design and participants

We conducted a phase III, open-label, parallel-group, randomised controlled clinical trial in western Kenya. The participants were recruited from 16 primary schools in Homabay and Migori Counties, Kenya. These included four schools in the Rachuonyo sub-county, Homabay, and 12 schools in the Nyatike and Suna West sub-counties, Migori. The schools were selected because of their proximity to previously identified schistosomiasis transmission sites. The study area has year-round malaria transmission and is endemic to *S. mansoni* with foci of *S. haematobium* [28–30]. The study was approved by the ethics review committee of the Kenya Medical Research Institute (KEMRI SSC # 2504).

Before starting the study, we met with parents, teachers, and community leaders to explain the study’s objectives, procedures, and potential benefits and risks. Written informed consent was obtained from parents or legal guardians of the eligible children. All explanations and informed consent procedures were performed in the primary language of the parents or guardians. The children were invited to give informed assent for screening and enrolment by writing their names and ticking a box with the following statement: "I agree to participate in this study".

The children were assessed for infection with *S. mansoni* or *S. haematobium*. Children who tested positive for *S. mansoni* or *S. haematobium* infection were eligible to participate in the study if they were 7–15 years old, were in grades four to six, and could take oral medication. We excluded children who weighed > 50 kg, were pregnant, had evidence of infection with *Plasmodium falciparum* or other *Plasmodium* spp., had severe illness (such as epilepsy), had signs of severe malnutrition, had used an anti-malaria or anti-schistosomal drug within 28 days of the study, or had a known history of hypersensitivity to artesunate, sulfonamide, or praziquantel agents.

### Randomisation and masking

Children with parasitologically confirmed *S. mansoni* or *S. haematobium* infection were randomly assigned (1:1:1) to receive praziquantel alone, artesunate plus sulfalene-pyrimethamine, or a combination of the two. This was performed using a computer-generated stratified block randomisation list provided by an independent statistician. The randomisation sequence was stratified by schistosome species (*S. mansoni* or *S. haematobium*) in blocks of nine. The study nurse administered the treatment, and the participants were aware of the treatment group assignment. However, laboratory technicians and clinicians assessing the study outcomes were blinded to treatment assignments throughout the study.

### Procedures

#### Laboratory procedures

Each child was given plastic containers labelled with unique screening identification numbers and instructed to provide fresh stool and urine samples. The samples were transferred in cooler boxes to a nearby study laboratory in Migori or Homabay for processing. For *S. mansoni*, duplicate slides were prepared from the stool samples and independently examined under a microscope by two experienced laboratory technicians. *Schistosoma mansoni* and soil-transmitted helminth eggs were quantified using the Kato-Katz technique, with a template containing approximately 41.7 mg of faeces when filled [31]. Eggs of soil-transmitted infections, such as *Trichuris trichiura*, *Ascaris lumbricoides*, and hookworm, were also assessed and recorded for each species separately. The number of *S. mansoni* eggs per slide was counted, and the arithmetic mean from the two slides was multiplied by 24 to express the number of eggs per gram (epg) of faeces. The intensity of the infection was categorised based on the WHO classification as light (1–99 epg), moderate (100–399 epg), or heavy (≥ 400 epg) [32].

For *S. haematobium*, a fresh urine sample was collected between 1000 and 1400 h after rigorous exercise (to enable maximum egg excretion). Haematuria and proteinuria were tested using dipsticks. Quantitative analysis of urine was performed using the urine filtration technique. The urine sample was vigorously shaken, and 10 ml was filtered through a 13-mm filter with an aperture of 12 µm (Sterlitech Corp., Auburn, WA, USA). The filters were placed on microscope slides, a drop of iodine was added, and the slides were read independently under a microscope by two laboratory technicians. The number of *S. haematobium* eggs was counted and expressed as eggs per 10 ml urine. The intensity of the infection was categorised as light (1–49 ep/10 ml) or heavy (≥ 50 ep/10 ml) based on the WHO classification [32].

As a quality control measure for interobserver variability, a third technician re-read a random selection of 10% of slides and all slides for which the readings varied by > 20% between the two technicians.

A capillary blood sample was taken by a fingerprick to test for malaria and measure haemoglobin. Haemoglobin was measured using a portable hemoglobinometer (HemoCue 301, Angelholm, Sweden). We used rapid diagnostic test strips (Paracheck, Orchid Biomedical Systems, India) to assess the presence of malaria antigens.

#### Participant screening and recruitment

The study clinician assessed the children for eligibility through a brief medical history and clinical examination. Children who tested positive for *S. mansoni* or *S. haematobium* and met all eligibility criteria were invited to participate in the study. The enrolling clinician took a standard baseline medical history and clinically examined the children, including weight (using a digital weighing scale) and height (using a Seca 213 portable stadiometer) measurements. The clinician also assessed the size of the liver and spleen. The study clinicians sequentially assigned study numbers to eligible children at enrolment.

#### Treatment and follow-up

The study nurse administered the study drugs according to the randomisation sequence. All children received food items (orange juice and slices of bread) to reduce the nauseating effect of the study drugs and improve the drug bioavailability before drug ingestion [33]. The study included three treatment groups. Children assigned to the praziquantel group received 40 mg/kg of praziquantel (Biltricide, Bayer Healthcare, Leverkusen, Germany) to the nearest half tablet (600 mg). Children assigned to the artesunate plus sulfalene-pyrimethamine group received a single oral dose of artesunate plus sulfalene-pyrimethamine (12 mg/kg of the artesunate component) administered as one tablet for those weighing 15 ≤ 29.9 kg, two tablets for 30 ≤ 44.9 kg, and three tablets for 45–50 kg. Artesunate plus sulfalene-pyrimethamine (Coarinate Adult, Dafra Pharma, Turnhout, Belgium) is a co-formulated fixed-dose combination tablet consisting of artesunate (200 mg), sulfamethoxypyrazine (500 mg), and pyrimethamine (25 mg). Children assigned to combination therapy received one dose of 40 mg/kg praziquantel and a single dose of 12 mg/kg artesunate plus sulfalene-pyrimethamine (one tablet for 15 ≤ 29.9 kg, two tablets for 30 ≤ 44.9 kg, and three tablets for 45–50 kg).

All study drugs were given orally by the study nurse, and the children were observed for 3 h after taking the drug to ensure retention and to check for any immediate adverse events. If vomiting occurred within an hour of drug ingestion, a second total dose was given. Children who experienced repeated vomiting were withdrawn from the study. According to national treatment guidelines, all children with helminthic infections were treated with a single dose of 400 mg of albendazole.

All children were followed up for a total of 6 weeks. During the week 6 follow-up visit, the children provided a stool and urine sample to be assessed for schistosome eggs, as described above. Participants who did not return for the scheduled follow-up visit were visited at home. At the end of the study, all the children were treated with a single oral dose of 40 mg/kg praziquantel.

### Outcomes

The primary efficacy endpoints were the cure and egg reduction rates at week 6 post-treatment. The cure rate was defined as the proportion of study participants not excreting schistosome eggs in stool or urine at week 6 after study treatment. The cure rate was computed separately for children diagnosed with *S. mansoni* or *S. haematobium* at enrolment. The egg reduction rate (ERR) was calculated from the arithmetic mean count and defined as the proportional reduction in the number of schistosome eggs in the stool or urine samples from baseline to post-treatment. The ERR was computed according to the following formula:

$$\left( {1 - \left[ {1 - \frac{{\text{arithmetic}\; \text{mean} \; \text{egg}\; \text{count}\; \text{after}\; \text{treatment}}}{{\; \text{arithmetic}\; \text{mean}\; \text{egg}\; \text{count}\; \text{at}\; \text{enrollment}}}} \right]} \right)*100$$

Secondary efficacy endpoints included the proportion of participants cured by week 6, according to the infection intensity at enrolment. The safety endpoint was the incidence of adverse events in each treatment arm. An adverse event was defined as a sign, symptom, intercurrent illness, or abnormal laboratory result that was not present at enrolment but occurred within 3 h of study drug administration. A serious adverse event was defined as an adverse event that was lethal, life-threatening, disabling, or required hospital admission. Within 3 h of ingesting the study treatment, the clinician evaluated adverse events and classified them as mild, moderate, or severe, depending on their severity and impact on daily activities.

### Statistical analysis

With 80% power and a two-sided error of 5%, we calculated that 136 children would be needed in each treatment arm to detect a statistically significant difference in cure rates, assuming a cure rate of 72% with praziquantel [34] and 84% with combination therapy [35]. After an additional 14 (10%) children per treatment arm were included to allow for loss to follow-up, we computed a total sample size of 450 children (150 per treatment arm). For each treatment group, we stratified the sample size at a ratio of 2:1 by schistosome species (*S. mansoni* to *S. haematobium*).

The data collected from the participants were recorded on paper-based case report forms, entered into computers using MS Access, cross-checked, and analysed with IBM SPSS for Windows version 20 (SPSS Inc, Chicago, IL) and STATA version 12.0 (StataCorp.; College Station, TX, USA). Baseline characteristics and outcome data were analysed for all participants who received at least one dose of the study drug, whether they completed the study or not. The final analysis included participants who had received study medication, had at least one stool or urine sample examined at follow-up, and were not withdrawn or excluded for any reason (available case analysis).

For each treatment group, cure rates were computed (separately for children with *S. mansoni* and *S. haematobium* at enrolment) as the proportion of randomised participants who were cured by week 6 post-treatment. We used Pearson’s chi-square test for contingency tables to compare cure rates between treatment groups (in an available case population) and summed them up as risk ratios (RRs) with 95% confidence intervals. The risk ratio was computed as the proportion of children cured in the artesunate plus sulfalene-pyrimethamine group or the combination therapy group divided by the proportion of children cured in the praziquantel group. The arithmetic mean egg count was calculated for all participants excreting schistosome eggs at enrolment and follow-up, and the reduction in egg count was expressed as a percentage. Two-sided *p*-values < 0.05 were considered to indicate statistical significance.

## Results

### Participant flow and baseline characteristics

**Fig. 1:**
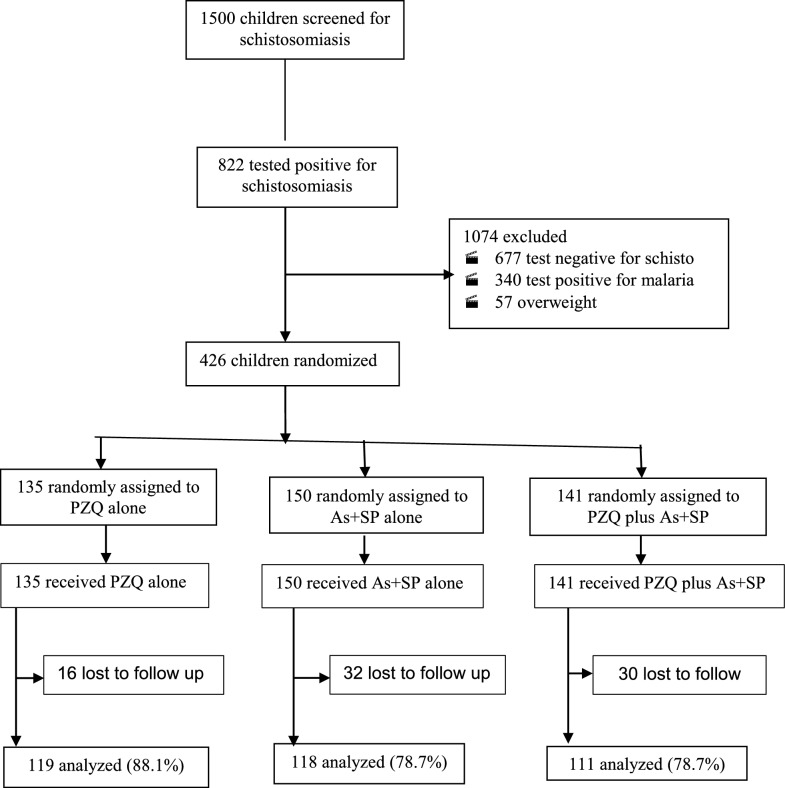
Trial profile

Between September 11, 2014, and October 25, 2015, 1500 children were invited to participate in the study. Overall, 822 (54.8%) children tested positive for schistosomiasis, of whom 166 (11.1%) had *S. haematobium*, 513 (34.2%) had *S. mansoni*, and 143 (9.5%) had mixed infections (i.e. both *S. haematobium* and *S. mansoni*). A total of 1074 children were excluded from the trial, including 340 (22.6%) children who tested positive for malaria. A total of 426 children were enrolled, 135 of whom were randomly assigned to receive praziquantel, 150 to receive artesunate plus sulfalene-pyrimethamine, and 141 to receive combination therapy (praziquantel combined with artesunate plus sulfalene-pyrimethamine). Figure 1 shows the trial profile.

The baseline characteristics assessed were balanced between treatment groups at enrolment. A total of 319 (74.9%) children were recruited from Migori County; 222 (52.1%) were boys; 335 (78.6%) had *S. mansoni* and 145 (34.0%) had *S. haematobium* infection. Fifty-four (12.7%) of those enrolled had mixed schistosome infections: 17 were assigned to praziquantel alone, 20 to artesunate plus sulfalene-pyrimethamine, and 17 to combination therapy. At week 6, 78 (18.3%) children were lost to follow-up and were unavailable for analysis: 16 from the praziquantel alone group, 32 from the artesunate plus sulfalene-pyrimethamine group, and 30 from the combination therapy group. A total of 348 (81.7%) children were available for analysis; 119 (88.1%) were in the praziquantel alone group, 118 (78.7%) were in the artesunate plus sulfalene-pyrimethamine group, and 111 (78.7%) were in the combination therapy group. Overall, 260 (77.6%) of those analysed had *S. mansoni*, while 133 (91.7%) had *S. haematobium* at enrolment. Table 1 is a summary of the participant characteristics at baseline.

**Table 1:** Baseline characteristics of treatment groups

Variable	PZQ alone	As + SMP	PZQ plus As + SMP
*Schistosoma mansoni*-infected children			
Number	106	119	110
Sex			
Male/female	56/50	64/55	51/59
Age (years)			
Mean (SD), median	12.08 (1.57), 12.0	12.03 (1.48), 12.0	11.86 (1.46), 12.0
County, N (%)			
Homabay	25 (23.6)	17 (14.3)	23 (20.9)
Migori	81 (76.4)	102 (85.7)	87 (79.1)
Body weight (kg)			
Mean (SD), median	37.1 (5.91), 37.0	37.5 (6.93), 37.4	36.3 (6.48), 36.0
Haemoglobin (g/dl)			
Mean (SD), median	12.7 (1.61), 12.9	12.8 (1.41), 12.9	12.4 (2.14), 12.7
Infection intensity, N (%)			
Light (1–99 epg)	69 (65.1)	80 (67.2)	67 (60.9)
Moderate (100—399 epg)	32(30.2)	31 (26.1)	32 (29.1)
Heavy (≥ 400 epg)	5 (4.7)	8 (6.7)	11 (10.0)
*Schistosoma haematobium*-infected children			
Number	46	51	48
Sex			
Male/female	25/21	33/18	20/28
Age (years)			
Mean (SD), median	12.04 (1.30), 12.0	12.04 (1.50), 12.0	11.98 (1.35), 12.0
County, N (%)			
Homabay	11 (23.9)	20 (39.2)	16 (33.3)
Migori	35 (76.1)	31 (60.8)	32 (66.7)
Body weight (kg)			
Mean (SD), median	36.7 (6.06), 35.0	36.6 (7.45), 35.0	38.2 (6.73), 37.8
Haemoglobin (g/dl)			
Mean (SD), median	12.8 (1.77), 12.8	12.5 (1.61), 12.7	12.3 (2.07), 12.9
Infection intensity, N (%)			
Light (1–49 ep/10 ml)	35 (76.1)	41 (80.4)	43 (89.6)
Heavy (≥ 50 ep/10 ml)	11 (23.9)	10 (19.6)	5 (10.4)

*PZQ* praziquantel, *As + SMP* artesunate plus sulfalene-pyrimethamine, *SD* standard deviation, *CI* confidence interval

### Effect of treatment on *S. mansoni* infection

A total of 185 (71.2%) of the available 260 children were cured, while 75 (28.8%) were still parasitaemic. The cure rates were 75.6%, 60.7%, and 77.8% in children who received praziquantel alone, artesunate plus sulfalene-pyrimethamine, and combination therapy, respectively. A significantly lower proportion of children in the artesunate plus sulfalene-pyrimethamine group was cured than in the praziquantel alone group (*p* = 0.032). A similar number of children on combination therapy was cured as those on praziquantel alone (*p* = 0.732). The cure rate in all three treatment groups decreased as the infection intensity increased. However, the proportion of cured children was significantly greater among those with light-intensity *S. mansoni* infections at enrolment than among those with moderate- or heavy-intensity infections. Of those not cured, 30/75 had light-intensity infections, 37/75 had moderate-intensity infections, and 8/75 had heavy-intensity infections. The egg reduction rates at week 6 were 80.1%, 85.0%, and 88.4% in children who had received praziquantel alone, artesunate plus sulfalene-pyrimethamine, and combination therapy, respectively. See Table 2.

**Table 2:** Efficacy endpoints for *Schistosoma mansoni*-infected participants

Variable	PZQ Alone	As + SP alone	PZQ plus As + SP
Cure rate at 6 weeks (%) [95% CI]	68/90 (75.6) [66.7–84.5]	54/89 (60.7) [50.6–70.8]	63/81(77.8) [68.7–86.9]
Risk ratio (95% CI)	1	0.80 (0.65–0.99)	1.02 (0.87–1.21)
*P* value	Ref	0.032	0.732
Cure rate by initial infection intensity			
*Light infection,* (n/N (%)	50/55 (90.9)	43/61 (70.5)	44/51 (86.3)
*Moderate infection,* n/N (%)	15/30 (50.0)	6/21 (28.6)	15/22 (68.2)
*Heavy infection,* (n/N (%)	3/5 (60)	5/7 (71.4)	4/8 (50.0)
Arithmetic mean EPG (95% CI)			
Before treatment	128.15 (87.63–168.68)	158.22 (90.09–226.34)	144.98 (106.97–182.99)
After treatment	25.47 (–6.44–57.38)	23.73 (13.39–34.08)	16.89 (7.12–26.65)
Egg reduction rate * (95% CI)	80.1 (71.8–88.3)	85.0 (77.3–92.2)	88.4 (81.4–95.4)

*PZQ* praziquantel, *As + SMP* artesunate plus sulfalene-pyrimethamine, *Ref* reference, *CI* confidence interval

*Data presented as percent

### Effect of treatment on *S. haematobium* infection

A total of 104 (78.2%) of the 133 available children were cured, while 29 (21.8%) were still parasitemic. The cure rates were 81.4%, 71.1%, and 82.2% in those who received praziquantel alone, artesunate plus sulfalene-pyrimethamine, and combination therapy, respectively. There was no significant difference in the cure rate between the artesunate plus sulfalene-pyrimethamine or combination therapy group and the praziquantel-only group. The cure rates did not decrease with increasing infection intensity except in the artesunate plus sulfalene-pyrimethamine group. Of those who were not cured, 16/29 had light-intensity infections, and 13/29 had heavy-intensity infections. The egg reduction rates at week 6 were 95.6%, 97.1%, and 97.7% in children who had received praziquantel alone, artesunate plus sulfalene-pyrimethamine, and combination therapy, respectively. See Table 3.

**Table 3:** Efficacy endpoints for *Schistosoma haematobium*-infected participants

Variable	PZQ Alone	As + SP alone	PZQ plus As + SP
Cure rate at 6 weeks (%) [95%CI]	35/43 (81.4) [69.8 to 93.0]	32/45 (71.1) [57.9 to 84.3]	37/45 (82.2) [71.0 to 93.4]
Risk ratio (95%CI)	1	0.87 (0.69 to 1.10)	1.01 (0.83 to 1.23)
*P* value	Ref	0.258	0.920
Cure rate by infection intensity			
*Light infection,* n/N (%)	27/33 (81.8)	31/36 (86.1)	34/41 (82.9)
*Heavy infection,* (n/N (%)	8/10 (80.0)	1/9 (11.1)	3/4 (75)
Arithmetic mean EP/10 ml (95%CI)			
Before treatment	50.22 (17.41 to 83.02)	47.25 (19.66 to 74.84)	35.10 (9.30 to 60.91)
After treatment	2.21 (-0.705 to 5.12)	1.38 (0.497 to 2.26)	0.822 (-0.099 to 1.74)
Egg reduction rate* (95%CI)	95.6 (89.7 to 100)	97.1 (92.5 to 100)	97.7 (93.5 to 100)

PZQ = praziquantel; As + SMP = artesunate plus sulfalene-pyrimethamine; Ref = reference; CI = confidence interval; *data presented as percent

### Effect of treatment on safety outcomes

Of the 426 children enrolled, 71 (16.7%) children reported an adverse event; 25 (5.9%), 19 (4.5%), and 27 (6.3%) were among those who had received praziquantel alone, artesunate plus sulfalene-pyrimethamine, and combination therapy, respectively. Overall, 221 adverse events were reported: 61 (27.6%) in the praziquantel alone group, 58 (26.2%) in the artesunate plus sulfalene-pyrimethamine group, and 112 (46.2%) in the combination therapy group. All reported adverse events were mild and resolved within 24 h after treatment. None of the reported adverse events required treatment. No serious adverse events were reported. The most common adverse events were abdominal pain, nausea, vomiting, and dizziness. See Table 4.

**Table 4:** Adverse events reported by the study participants

Adverse event	PZQ alone	As + SP alone	PZQ plus As + SP	Total
Weakness of body	8	7	11	26
Nausea	9	6	14	29
Vomiting	9	8	17	34
Headache	9	8	8	25
Diarrhea	3	4	5	12
Dizziness	12	14	12	38
Body rash	4	2	4	10
Skin itchiness	3	1	4	8
Abdominal pain	4	8	27	39
Number of adverse events reported, N (%)	61 (27.6%)	58 (26.2%)	102 (46.2%)	**221**
Number of children reporting adverse events, N (%)	25 (5.9%)	19 (4.5)	27 (6.3%)	71 (16.7%)

PZQ = praziquantel; As + SMP = artesunate plus sulfalene-pyrimethamine; SD = standard deviation; CI = confidence interval

## Discussion

We assessed the role of single-dose combination therapy, comprising praziquantel and artesunate plus sulfalene-pyrimethamine compared to praziquantel alone in treating African children with intestinal (*S. mansoni*) or urinary (*S. haematobium*) schistosomiasis. In general, the cure and egg reduction rates, assessed at 6 weeks, were relatively higher after treatment of *S. haematobium*-infected children than after treatment of *S. mansoni*-infected children. However, the cure rates with combination therapy were comparable to that of praziquantel in the treatment of children with either intestinal or urinary schistosomiasis. A single oral dose of artesunate plus sulfalene-pyrimethamine had a similar cure rate to praziquantel against urinary schistosomiasis but was inferior against intestinal schistosomiasis. Treatment of children with *S. haematobium* resulted in egg reduction rates > 90% in all three treatment groups, but these rates were < 90% in treating intestinal schistosomiasis. There was no difference in the cure or egg reduction rates after treatment of children with mixed infections (data not shown). A similar number of children in the three treatment groups experienced adverse events. The study drugs were well tolerated and no serious adverse events were reported.

At our study site, the prevalence of schistosomiasis in school-aged children was 55%, with most children presenting with light-intensity infections, including 10% with mixed schistosome infections. A single oral dose of praziquantel cured 75.6% and 81.4% of children with *S. mansoni* and *S. haematobium* infection, respectively. The egg reduction rates for children with *S. mansoni* and *S. haematobium* were 80.1% and 95.6%, respectively. These cure and egg reduction rates are consistent with a meta-analysis of previous studies [36]. The WHO recommends a 90% egg reduction rate for satisfactory efficacy in treating schistosomiasis [37]. The egg reduction rates after treatment of *S. mansoni* using praziquantel alone were < 90%, suggesting reduced praziquantel efficacy in our setting according to the WHO criteria. By contrast, the egg reduction rates were > 90% for *S. haematobium*, suggesting that praziquantel is still effective against *S. haematobium* in our setting. We noted a greater cure rate in children with light-intensity *S. mansoni* infections than in those with *S. haematobium* infections. This finding is consistent with the observation that the cure rate after a single dose of praziquantel is inversely proportional to the infection intensity at baseline [8, 38, 39].

Artesunate plus sulfalene-pyrimethamine is an excellent antimalarial drug that is effective as a 24-h or 3-day regimen for treating uncomplicated *P. falciparum* malaria [40, 41]. We found that a single oral dose of artesunate plus sulfalene-pyrimethamine had comparable efficacy to praziquantel against urinary schistosomiasis but was inferior against intestinal schistosomiasis. In previous studies, artesunate plus sulfalene-pyrimethamine was less effective than praziquantel in curing children with *S. mansoni* (14% vs 65%, 3-day regimen) or *S. haematobium* (44% vs 53%, 24-h regimen) [21, 22]. The current findings are consistent with a recent exploratory study in western Kenya where cure rates of 69% vs 80% were reported after single doses of artesunate plus sulfalene-pyrimethamine and praziquantel were compared in the treatment of children with *S. mansoni* infection [42]. Overall, the findings suggest a dose-response function indicating that a 3-day treatment regimen of 4 mg/kg of artesunate plus sulfalene-pyrimethamine (similar to the treatment of malaria) may be ineffective for curing *S. mansoni* infection. In this study, a single-dose artesunate plus sulfalene-pyrimethamine had a comparable cure and egg reduction rate to praziquantel against *S. haematobium* infection. For unclear reasons, our results contrast with those of a study in Mali, where a similar treatment regime cured only 43.9% of children with *S. haematobium* [21].

The combination of artemisinin derivatives with praziquantel is a novel approach for enhancing schistosomiasis control by leveraging drugs with different killing mechanisms. In this study, a single oral dose of combination therapy (praziquantel combined with artesunate plus sulfalene-pyrimethamine) did not improve treatment efficacy compared to praziquantel for either *S. mansoni* or *S. haematobium* infection. This finding contrasts a recent study in which a 3-day course of treatment with praziquantel plus dihydroartemisinin-piperaquine had superior efficacy to a single dose of praziquantel against *S. mansoni* infection in Tanzania [26]. However, our study is consistent with an exploratory trial in Cote d’Ivoire where comparable cure and egg reduction rates were observed when a 3-day course of praziquantel plus artesunate-mefloquine was compared to a single dose of praziquantel in the treatment of children with *S. haematobium* infection [27]. In China, comparable cure and egg reduction rates were observed when a 3-day course of artemether plus praziquantel was compared with a single dose of praziquantel in the treatment of *S. japonicum* [43]. The resolution of these inconsistent results calls for an urgent head-to-head comparison study of praziquantel plus the different artemisinin-based combinations with praziquantel alone to determine the “best” artemisinin-based combination to combine with praziquantel for schistosomiasis control. In general, we observed relatively higher cure and egg reduction rates after combination therapy for *S. haematobium* than for *S. mansoni* infection. These differences in response rates can be explained by the high proportion of light *S. haematobium* infection intensity at enrolment and the differential sensitivity of the schistosome species to treatment.

The study drugs were well tolerated, and most adverse events were mild and transient. This study is consistent with previous studies that reported only mild adverse events with either praziquantel or artesunate plus sulfalene-pyrimethamine. The most frequent adverse events across the treatment groups were abdominal pain, nausea, vomiting, and dizziness, which is consistent with previous studies [39, 44–47]. However, participants in the combination therapy arm reported a slightly higher number of adverse events than those in the other two treatment arms. Drug-drug interactions resulting from simultaneous drug administration and increased pill burden could explain the enhanced risk of adverse events in the combined therapy group. The use of self-reports, limited sample size, and study duration limited our ability to confidently compare drug safety. The relative safety of the drugs when administered separately suggests that sequential rather than simultaneous administration may improve the efficacy and safety outcomes.

Our study had several limitations. Most of our participants had light-intensity infections at baseline, meaning that these findings should be applied cautiously to high transmission areas since light-intensity infections are associated with high cure rates [8, 39, 40]. A double-blind design would have improved our study. However, incorporating matching placebo tablets for the drugs assessed in this study would have been impractical. We tested single stool and urine samples during enrolment and follow-up for logistical reasons. Kato-Katz and urine filtration tests for schistosomiasis have low sensitivity to low-intensity infections and may have misclassified some participants. It is unclear whether 6 weeks following therapy is the best time to assess treatment efficacy in schistosomiasis treatment studies [48]. In the absence of standardized treatment efficacy testing protocols, most studies evaluating anti-schistosomal drugs have assessed efficacy between 3 to 12 weeks post-treatment, making comparisons difficult. In this study, compliance was improved by the use of directly observed single-dose treatment. However, the assessment of kidney and liver functions would have improved our drug safety evaluation.

If efficacious, safe, acceptable, and feasible, the introduction of combination therapy may shift schistosomiasis control from morbidity to transmission control and hasten the global elimination agenda. Combined therapy may be effective in malaria-free settings, in individuals with both malaria and schistosomiasis, such as chemoprophylaxis for travellers to endemic areas, as a second-line treatment in the event of praziquantel failure and as seasonal chemoprophylaxis in malaria-endemic areas. Combination therapy is expensive and challenging to implement for mass drug administration. Additional studies are needed to validate these findings in different epidemiological settings, to assess other ACT-praziquantel combinations and drug-drug interactions, to evaluate appropriate dosing intervals, and to determine the impact of sequential or simultaneous delivery. Other studies are needed to monitor the markers of antimalarial drug resistance and the beneficial impact of ACT for malaria treatment on schistosomiasis control.

## Conclusions

In conclusion, the role of combination therapy in treating and controlling schistosomiasis remains unclear. In this study, combination therapy cured a high proportion of children with *S. haematobium* but overall it did not improve treatment efficacy for urinary or intestinal schistosomiasis. Sequential administration of praziquantel and artesunate plus sulfalene-pyrimethamine may improve the efficacy and safety outcomes of treatment.

## Data Availability

The datasets that were analysed for this study have beed submitted to the Infectious Disease Data Observatory (ID SHBUPQL).

## Abbreviations

ACT: Artemisinin-based combination therapy
As + SMP: Artesunate plus sulfalene-pyrimethamine
CI: Confidence interval
EPG: Eggs per gram
ERR: Egg reduction rate
Hb: Haemoglobin
KEMRI: Kenya Medical Research Institute
RR: Relative risk
PZQ: Praziquantel
SD: Standard deviation
SSC: Scientific steering committee
WHO: World Health Organisation
